# Machine learning for the prediction of acute kidney injury in patients with sepsis

**DOI:** 10.1186/s12967-022-03364-0

**Published:** 2022-05-13

**Authors:** Suru Yue, Shasha Li, Xueying Huang, Jie Liu, Xuefei Hou, Yumei Zhao, Dongdong Niu, Yufeng Wang, Wenkai Tan, Jiayuan Wu

**Affiliations:** 1 Clinical Research Service Center, The Affiliated Hospital of Guangdong Medical University, Zhanjiang, 524001 Guangdong Province China; 2 Collaborative Innovation Engineering Technology Research Center of Clinical Medical Big Data Cloud Service in Medical Consortium of West Guangdong Province, The Affiliated Hospital of Guangdong Medical University, Zhanjiang, 524001 Guangdong Province China; 3 Department of Gastroenterology, The Affiliated Hospital of Guangdong Medical University, Zhanjiang, 524001 Guangdong Province China

**Keywords:** Acute kidney injury, Sepsis, Machine learning, Prediction model, MIMIC- III database

## Abstract

**Background:**

Acute kidney injury (AKI) is the most common and serious complication of sepsis, accompanied by high mortality and disease burden. The early prediction of AKI is critical for timely intervention and ultimately improves prognosis. This study aims to establish and validate predictive models based on novel machine learning (ML) algorithms for AKI in critically ill patients with sepsis.

**Methods:**

Data of patients with sepsis were extracted from the Medical Information Mart for Intensive Care III (MIMIC- III) database. Feature selection was performed using a Boruta algorithm. ML algorithms such as logistic regression (LR), *k*-nearest neighbors (KNN), support vector machine (SVM), decision tree, random forest, Extreme Gradient Boosting (XGBoost), and artificial neural network (ANN) were applied for model construction by utilizing tenfold cross-validation. The performances of these models were assessed in terms of discrimination, calibration, and clinical application. Moreover, the discrimination of ML-based models was compared with those of Sequential Organ Failure Assessment (SOFA) and the customized Simplified Acute Physiology Score (SAPS) II model.

**Results:**

A total of 3176 critically ill patients with sepsis were included for analysis, of which 2397 cases (75.5%) developed AKI during hospitalization. A total of 36 variables were selected for model construction. The models of LR, KNN, SVM, decision tree, random forest, ANN, XGBoost, SOFA and SAPS II score were established and obtained area under the receiver operating characteristic curves of 0.7365, 0.6637, 0.7353, 0.7492, 0.7787, 0.7547, 0.821, 0.6457 and 0.7015, respectively. The XGBoost model had the best predictive performance in terms of discrimination, calibration, and clinical application among all models.

**Conclusion:**

The ML models can be reliable tools for predicting AKI in septic patients. The XGBoost model has the best predictive performance, which can be used to assist clinicians in identifying high-risk patients and implementing early interventions to reduce mortality.

**Supplementary Information:**

The online version contains supplementary material available at 10.1186/s12967-022-03364-0.

## Introduction

Acute kidney injury (AKI) is a common and complex clinical complication in intensive care unit (ICU) settings [1]. In the ICU, approximately 53% of AKI is caused by sepsis and subsequently contributes to longer hospital stay, higher morbidity, and heavier financial burden to patients [2, 3]. Despite improvements in clinical treatment, the mortality of AKI remains unchanged and reaches as high as 40–44% in patients with sepsis due to multiorgan failure, microvascular dysfunction, and systemic inflammatory response syndrome [4–6]. However, AKI can be reversed at the early stage through timely intervention and effective treatment, thereby reducing AKI-related mortality [7]. Therefore, identifying patients with high risk of AKI is of vital importance for the management of patients with sepsis in ICU settings.

The prediction of AKI in patients with sepsis has always been a hot topic in critical care medicine. Some biomarkers, such as microRNA-22-3p [8], neutrophil gelatinase-associated lipocalin [9], procalcitonin [10], urinary miR-26b [11], and soluble thrombomodulin [12], have been reported to be associated with AKI in sepsis. However, they are difficult to popularize in clinical settings due to the high cost and requirement of testing technology. Some scoring systems, including acute physiology and chronic health evaluation-II, the simplified acute physiology score (SAPS) II, and sequential organ failure assessment (SOFA), have also been used in AKI prediction, but their performances are unsatisfactory due to poor specificity and sensitivity [13, 14]. In addition, some multivariate predictive models based on traditional statistical methods, such as logistic regression (LR) and Cox proportional risk model, have been developed for predicting the development of AKI among patients with sepsis. Fan et al. [15] applied LR to develop a prediction model for AKI in 15,726 patients with sepsis, and the model showed a preferable predictive accuracy. Importantly, the relationship between variables is complex, including linear or nonlinear relationship, which is prominent in ICU settings. However, LR is defaulted to handle the linear relationship between independent and dependent variables, and may oversimplify the complex nonlinear relationship. Moreover, LR is prone to be affected by multicollinearity between variables, which may reduce the performance of the model. Therefore, exploring more effective and accurate prediction tools is extremely important in the management of septic patients.

Recently, machine learning (ML) has attracted the attention of and gained recognition from clinicians due to the evolution of statistical theory and computer technology. Novel ML techniques have been widely used in predictive models of various diseases and show better performance compared with those of traditional LR or Cox regression analyses [16, 17]. We can find quite a few efforts on the application of ML algorithms for AKI prediction. For example, Chiofolo et al. [18] developed an AKI prediction model using automatic continuous random forest algorithm in critically ill patients, and achieved a preferable capability for early identification of high-risk patients. Le et al. [19] formulated a prediction system for AKI in the ICU settings using convolutional neural networks (CNNs), and found that the predictive performance of the CNN model outperformed that of SOFA scoring system. Lin et al. [20] found that random forest had greater potential in predicting mortality in patients with AKI rather than support vector machine (SVM), artificial neural network (ANN), and SAPS II. However, evidence showing the advantage of the ML algorithms in the prediction of AKI in septic patients is still lacking. In this study, we aimed to develop and validate multiple ML models to predict AKI in septic patients and to find the model with the best predictive performance.

## Methods

### Data source

Using the Structured Query Language, data of patients with sepsis were extracted from a single-center publicly available database called the Medical Information Mart for Intensive Care III (MIMIC-III) database [21]. The MIMIC-III database is an integrated, de-identified, comprehensive clinical dataset containing all patients admitted to the ICUs of Beth Israel Deaconess Medical Center in Boston, MA, from June 1, 2001, to October 31, 2012. The MIMIC-III includes detailed information about admitted and discharged patients, such as demographic characteristics, monitoring vital signs, laboratory and microbiological examination, imaging examination, observation and recording of intake and output, drug treatment, length of stay, survival data, and discharge or death records. To apply for access to the database, we passed the protection of human research participants examination and obtained the certificate (No. 9983480).

### Participants

When patients were diagnosed with sepsis using the International Classification of Disease 9th revision (ICD-9) (99591, 99592, 78552) after first ICU admission, the patient eligibility was considered. Then, the Kidney Disease: Improving Global Outcomes (KDIGO) criteria [22] were used to determine whether AKI occurred in patients with sepsis during hospitalization. Patients who left the ICU within 48 h, aged < 18 years old and > 89 years old, or previously had AKI or renal failure were excluded. Moreover, patients with missing > 20% individual data or receiving renal replacement therapy (RRT) or continuous RRT at admission were excluded.

### Data extraction

Patient data in the initial 24 h following admission were retrieved from the MIMIC- III database. The following information was used in this study: (1) demographic features, including sex, age, and ethnicity; (2) comorbidities, including congestive heart failure, hypertension, chronic pulmonary, diabetes, and liver disease; (3) vital signs, including heart rate, temperature, oxygen saturation (SpO_2_), systolic blood pressure (SysBP), and diastolic blood pressure (DiasBP); (4) laboratory parameters, including total bilirubin, anion gap, albumin, chloride, potassium, sodium, lactate, partial thromboplastin time (PTT), prothrombin time (PT), international normalized ratio (INR), creatinine, blood urea nitrogen (BUN), and glucose; (5) therapeutic and clinical managements, including mechanical ventilation and vasopressor use. For some variables with multiple measurements, we included the maximum and minimum values for analysis. For SOFA and SAPS-II scores, we only included the initial test values for analysis. Because this was an epidemiological study based on hypothesis, no attempt was made to estimate the sample size of the study. Instead, all eligible patients in the MIMIC- III database were enrolled to achieve a maximized statistical power.

In order to minimize the bias resulting from missing data, variables with over 20% missing values were excluded in the final cohort, and other variables were duplicated using multiple imputation (MI) method. MI is an excellent and widely used method in dealing with missing values [23]. MI can impute each missing value with multiple plausible possible values. This procedure takes into account uncertainty behind the missing value and can produce several datasets from which parameters of interest can be estimated [24]. For example, if you are interested in coefficient for a covariate in a multivariable model, the coefficients will be estimated from each dataset, resulting in multiple coefficients. Considering the uncertainty in the estimation of missing values, these coefficients are combined to give a valid estimate of the coefficient. The coefficient variance estimated by MI is less likely to be underestimated than that estimated by single imputation [25].

### Statistical analysis

Continuous variables were summarized as the median with interquartile range and were compared using the Wilcoxon rank-sum test. Categorial variables were expressed as number and percentage and were compared using the Chi-square tests or Fisher’s exact probability method.

Feature selection is an important step in model construction. The Boruta algorithm was used to identify the most important features by comparing the *Z*-value of each feature against that of “shadow features”. By duplicating all real features and shuffling them sequentially, the *Z*-value of each attribute is obtained from a random forest model in each iteration, and the *Z*-value of shadow is created by random shuffling of the real features. A real feature is regarded as “important” if its *Z*-value is greater than the maximal *Z*-value of shadow features in multiple independent trials [26]. After feature selection, seven ML algorithms, including LR, *k*-nearest neighbors (KNN), SVM, decision tree, random forest, extreme gradient boosting (XGBoost), and ANN, were employed for model construction. A tenfold cross-validation was applied for the training and validation sets to prevent overfitting, and it was also used to formulate predictive models. Accordingly, the whole dataset was randomly divided into 10 folds. Nine of them were used as the training set for model development, and the remaining one was used as the validation set for model validation. Because each of the 10 folds was used as the validation set, the above process was repeated 10 times. Finally, the performance of each model was validated and compared in the validation set. In our cases, the model with the highest area under curve (AUC) of the receiver operating characteristic (ROC) curve was selected as the optimal model of each algorithm. Because SOFA and SAPS II scores were used as common tools for predicting the illness severity and prognosis in critically ill patients, we also compared the predictive abilities of ML-based predictive models with those of the conventional scoring systems.

The performance of the predictive models was performed with respect to discrimination, calibration, and clinical utility. The discrimination was quantitatively evaluated by the AUC of the ROC curve, sensitivity, specificity, recall, accuracy, and F1 score. The calibration was visually assessed through the graphical representations of the consistency of the predictive probabilities and the observed outcomes based on 1000 bootstrap resamples. The clinical application was investigated by decision curve analysis (DCA). The statistical analyses and modeling process were conducted by using R version 4.0.5, and a two-sided *P*-value < 0.05 was regarded as statistically significant.

## Results

### Baseline characteristics

**Fig. 1:**
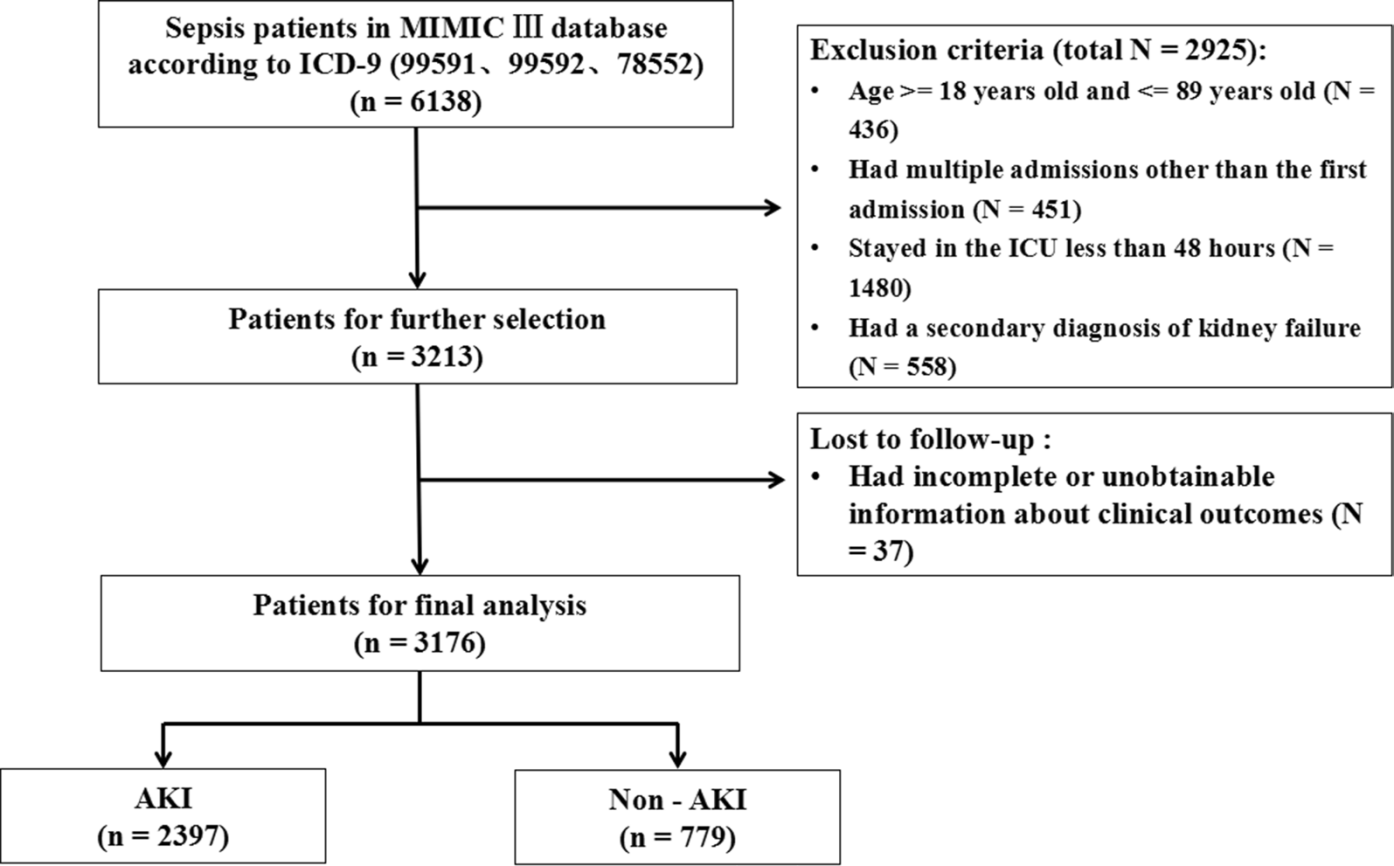
The flowchart of patient selection. MIMIC: Medical Information Mort for Intensive Care; ICU: intensive care unit

A total of 6138 patients were diagnosed with sepsis at admission according to ICD-9. Moreover, 2961 patients were excluded according to the exclusion criteria (Fig. 1). Finally, a total of 3176 patients were included in our analysis, of which 2397 patients (75.5%) had AKI after ICU admission.

The differences in characteristics between AKI and non-AKI groups are described in Table 1. Male patients were more likely to develop AKI than female patients during hospitalization. Patients who suffered from AKI had higher age and BMI; higher incidence of congestive heart failure, cardiac arrhythmias, hypertension, liver disease, paralysis, chronic pulmonary disease, diabetes, and coagulopathy; and higher rate of mechanical ventilation and vasopressor use compared with those without AKI. The maximum values of anion gap, albumin, bilirubin, creatinine, chloride, glucose, lactate, potassium, PTT, INR, PT, BUN, heart rate, temperature, and SpO_2_ and the minimum values of anion gap, albumin, bilirubin, creatinine, chloride, lactate, potassium, PTT, INR, PT, BUN, heart rate, temperature, SpO_2_, sodium, SysBP, and DiasBP were much higher in septic patients with AKI compared with those without AKI (*P* < 0.05). However, the levels of urine output and eGFR in the AKI group were lower than those in the non-AKI group (*P* < 0.05).

**Table 1:** Baseline characteristics of the patients with sepsis

Characteristics	Total (n = 3176)	Non-AKI (n = 779)	AKI (n = 2397)	*P* value
Male, n (%)	1756 (55.3%)	465 (59.7%)	1291 (53.9%)	0.004
Age (years)	66 (54–77)	62 (52–73)	67 (55–78)	< 0.001
Ethnicity, n (%)				0.147
White	2391 (75.3%)	566 (72.7%)	1825 (76.1%)	
Black	260 (8.2%)	71 (9.1%)	189 (7.9%)	
Other	525 (16.5%)	142 (18.2%)	383 (16.0%)	
BMI (kg/m^2^)	27 (23–32)	26 (23–30)	28 (23–32)	< 0.001
Congestive heart failure, n (%)	1034 (32.6%)	193 (24.8%)	841 (35.1%)	< 0.001
Cardiac arrhythmias, n (%)	1256 (39.5%)	222 (28.5%)	1034 (43.1%)	< 0.001
Hypertension, n (%)	1422 (44.8%)	317 (40.7%)	1105 (46.1%)	0.008
Paralysis, n (%)	125 (3.9%)	45 (5.8%)	80 (3.3%)	0.002
Chronic pulmonary, n (%)	766 (24.1%)	163 (20.9%)	603 (25.2%)	0.016
Diabetes, n (%)	865 (27.2%)	187 (24.0%)	678 (28.3%)	0.020
Liver disease, n (%)	626 (19.7%)	110 (14.1%)	516 (21.5%)	< 0.001
Coagulopathy, n (%)	830 (26.1%)	173 (22.2%)	657 (27.4%)	0.004
Mechanical ventilation, n (%)	1840 (57.9%)	262 (33.6%)	1578 (65.8%)	< 0.001
Vasopressor, n (%)	1990 (62.7%)	352 (45.2%)	1638 (68.3%)	< 0.001
Anion gap_min (mmol/l)	12 (11–14)	12 (10–14)	13 (11–14)	< 0.001
Anion gap_max (mmol/l)	16 (14–19)	15 (13–18)	16 (14–19)	< 0.001
Albumin_min (g/dl)	2.7 (2.3–3.1)	2.8 (2.4–3.1)	2.6 (2.2–3.1)	< 0.001
Albumin_max (g/dl)	2.8 (2.4–3.2)	2.8 (2.5–3.3)	2.8 (2.3–3.2)	0.002
Bilirubin_min (mg/dl)	0.7 (0.4–1.7)	0.6 (0.4–1.3)	0.7 (0.4–1.7)	< 0.001
Bilirubin_max (mg/dl)	0.8 (0.5–2.1)	0.7 (0.4–1.5)	0.9 (0.5–2.3)	< 0.001
Creatinine_min (mg/dl)	0.9 (0.7–1.4)	0.9 (0.7–1.2)	1 (0.7–1.4)	< 0.001
Creatinine_max (mg/dl)	1.2 (0.8–1.8)	1.1 (0.8–1.55)	1.2 (0.8–1.8)	< 0.001
Chloride_min (mEq/l)	103 (98–108)	103 (99–108)	103 (98–107)	0.003
Chloride_max (mEq/l)	109 (104–113)	109 (105–113)	108 (104–113)	0.004
Glucose_min (mg/dl)	104 (87–125)	104 (88–121.5)	104 (87–127)	0.76
Glucose_max (mg/dl)	156 (124–205)	146 (118.5–194)	158 (126–209)	< 0.001
Lactate_min (mmol/l)	1.5 (1.1–2.1)	1.4 (1–1.8)	1.5 (1.1–2.1)	< 0.001
Lactate_max (mmol/l)	2.4 (1.6–4.2)	2.1 (1.5–3.5)	2.5 (1.6–4.4)	< 0.001
Platelet_min (K/UL)	183 (109–278)	188 (120–283)	182 (106–276)	0.103
Platelet_max (K/UL)	229.5 (148.3–339)	229 (149–339)	230 (148–340)	0.673
Potassium_min (mEq/l)	3.6 (3.3–4)	3.6 (3.3–3.9)	3.6 (3.3–4)	0.005
Potassium_max (mEq/l)	4.4 (4–4.9)	4.2 (3.9–4.7)	4.4 (4–4.9)	< 0.001
PTT_min (seconds)	31.1 (27.1–36.8)	30.2 (26.8–35.3)	31.3 (27.2–37.3)	< 0.001
PTT_max (seconds)	35.7 (29.7–46.9)	34 (28.85–41.85)	36.3 (30–48.6)	< 0.001
INR_min	1.3 (1.2–1.6)	1.3 (1.1–1.5)	1.3 (1.2–1.6)	< 0.001
INR_max	1.4 (1.2–1.9)	1.4 (1.2–1.7)	1.5 (1.3–2)	< 0.001
PT_min (seconds)	14.7 (13.4–17)	14.3 (13.2–16.2)	14.8 (13.4–17.3)	< 0.001
PT_max (seconds)	15.8 (14–19.4)	15.2 (13.8–17.8)	16 (14.1–19.9)	< 0.001
Sodium_min (mEq/l)	136 (133–140)	137 (133–140)	136 (133–140)	0.028
Sodium_max (mEq/l)	140 (137–143)	140 (137–143)	140 (137–143)	0.216
BUN_min (mg/dl)	21 (14–35)	18 (12–30)	23 (14–37)	< 0.001
BUN_max (mg/dl)	26 (17–42)	23 (15–38)	27 (18–44)	< 0.001
WBC_min (K/UL)	10.6 (6.6–15.7)	10.9 (6.6–15.7)	10.6 (6.6–15.7)	0.647
WBC_max (K/UL)	15 (9.7–21.5)	14.9 (9.5–21.25)	15 (9.8–21.6)	0.403
HeartRate_min (beats/minute)	78 (67–90)	77 (65–89)	78 (67–90)	0.02
HeartRate_max (beats/minute)	114 (99–128)	110 (97–125.5)	115 (100–129)	< 0.001
SysBP_min (mmHg)	83 (75–92)	86 (78–95)	82 (73–91)	< 0.001
SysBP_max (mmHg)	140 (126–154)	138 (126–153)	140 (127–155)	0.06
DiasBP_min (mmHg)	41 (34–48)	42 (35–49)	40 (34–47)	< 0.001
DiasBP_max (mmHg)	80 (70.3–91)	81 (72–90)	80 (70–91)	0.706
Temperature_min (℃)	36.1 (35.6–36.7)	36.2 (35.8–36.7)	36.1 (35.6–36.6)	< 0.001
Temperature_max (℃)	37.7 (37.1–38.4)	37.7 (37.1–38.6)	37.6 (37.1–38.3)	0.006
SpO2_Min (%)	92 (89–95)	93 (90–95)	92 (89–95)	0.002
SpO2_Max (%)	100 (100–100)	100 (100–100)	100 (100–100)	0.003
Urine output (ml)	790 (422.3–1380)	1005 (512.5–1627.5)	745 (410–1280)	< 0.001
eGFR (ml/min/1.73m^2^)	75.2 (47.4–107.8)	87.4 (58.0–118.2)	70.4 (43.7–105.4)	< 0.001

*AKI* acute kidney injury, *BMI* body mass Index, * PT* prothrombin time, *PTT* partial thromboplastin time, *INR* International Normalized Ratio, *BUN* blood urea nitrogen, WBC white blood cell, *SpO2* oxygen saturation, *SysBP* systolic blood pressure, *DiasBP* diastolic blood pressure, *eGFR* estimated glomerular filtration rate

### Feature selection

**Fig. 2:**
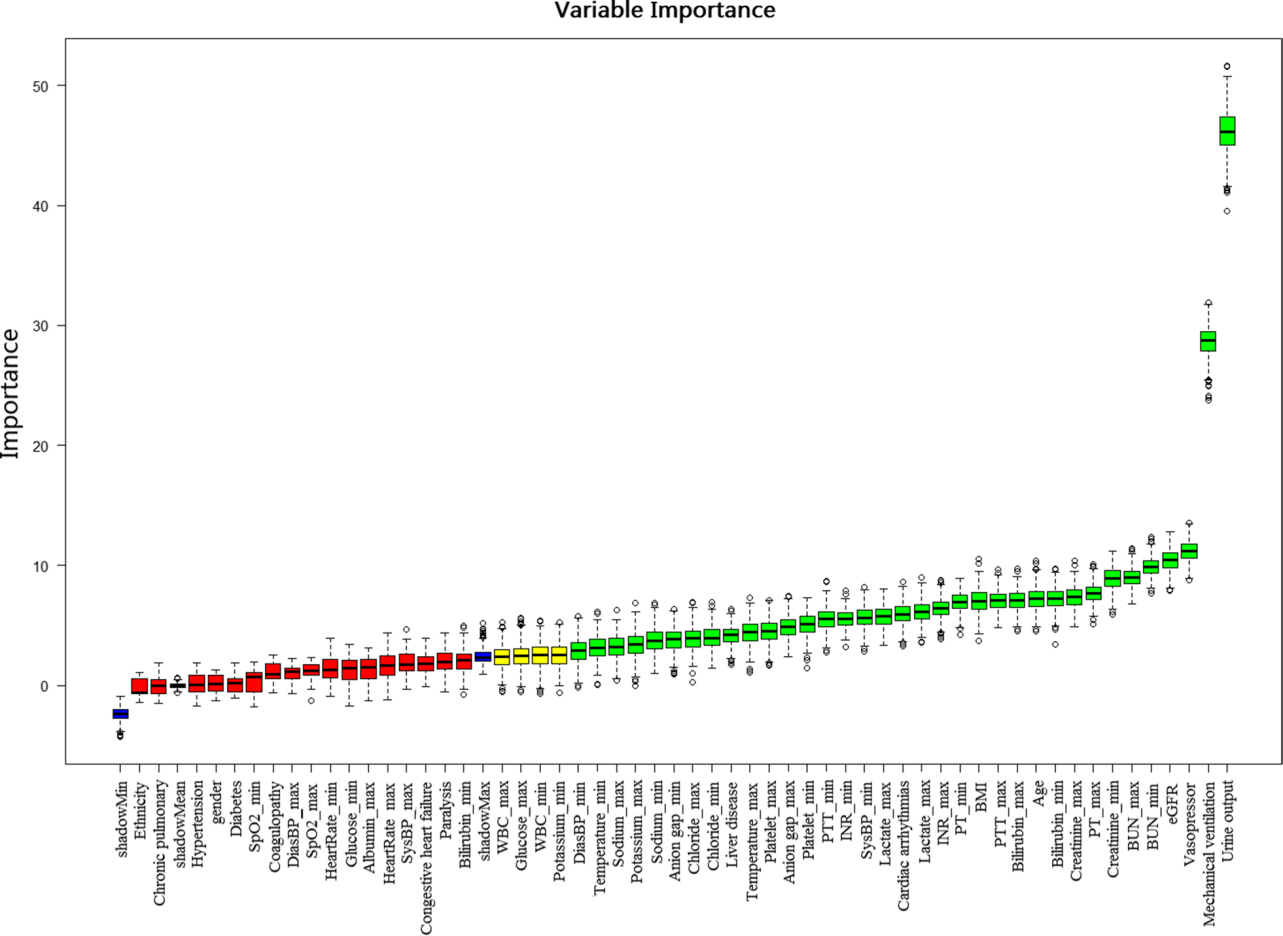
Feature selection based on the Boruta algorithm. The horizontal axis is the name of each variable, and the vertical axis is the *Z*-value of each variable. The box plot shows the *Z*-value of each variable during model calculation. The green boxes represent the first 35 important variables, the yellow represents tentative attributes, and the red represents unimportant variables. BMI: body mass Index; eGFR: estimated glomerular filtration rate; PT: prothrombin time; PTT: partial thromboplastin time; INR: International Normalized Ratio; BUN: blood urea nitrogen; SysBP: systolic blood pressure; DiasBP: diastolic blood pressure

The result of feature screening based on the Boruta algorithm is shown in Fig. 2. In order of Z-values, the 35 variables most closely associated with AKI were age, BMI, cardiac arrhythmias, liver disease, urine output, eGFR, mechanical ventilation, vasopressor, the maximum values of anion gap, bilirubin, creatinine, chloride, lactate, platelet, potassium, PTT, INR, PT, sodium, BUN, temperature, and the minimum values of anion gap, bilirubin, creatinine, chloride, lactate, platelet, PTT, INR, PT, sodium, BUN, temperature, SysBP, and DiasBP.

### Model performance comparisons

**Fig. 3:**
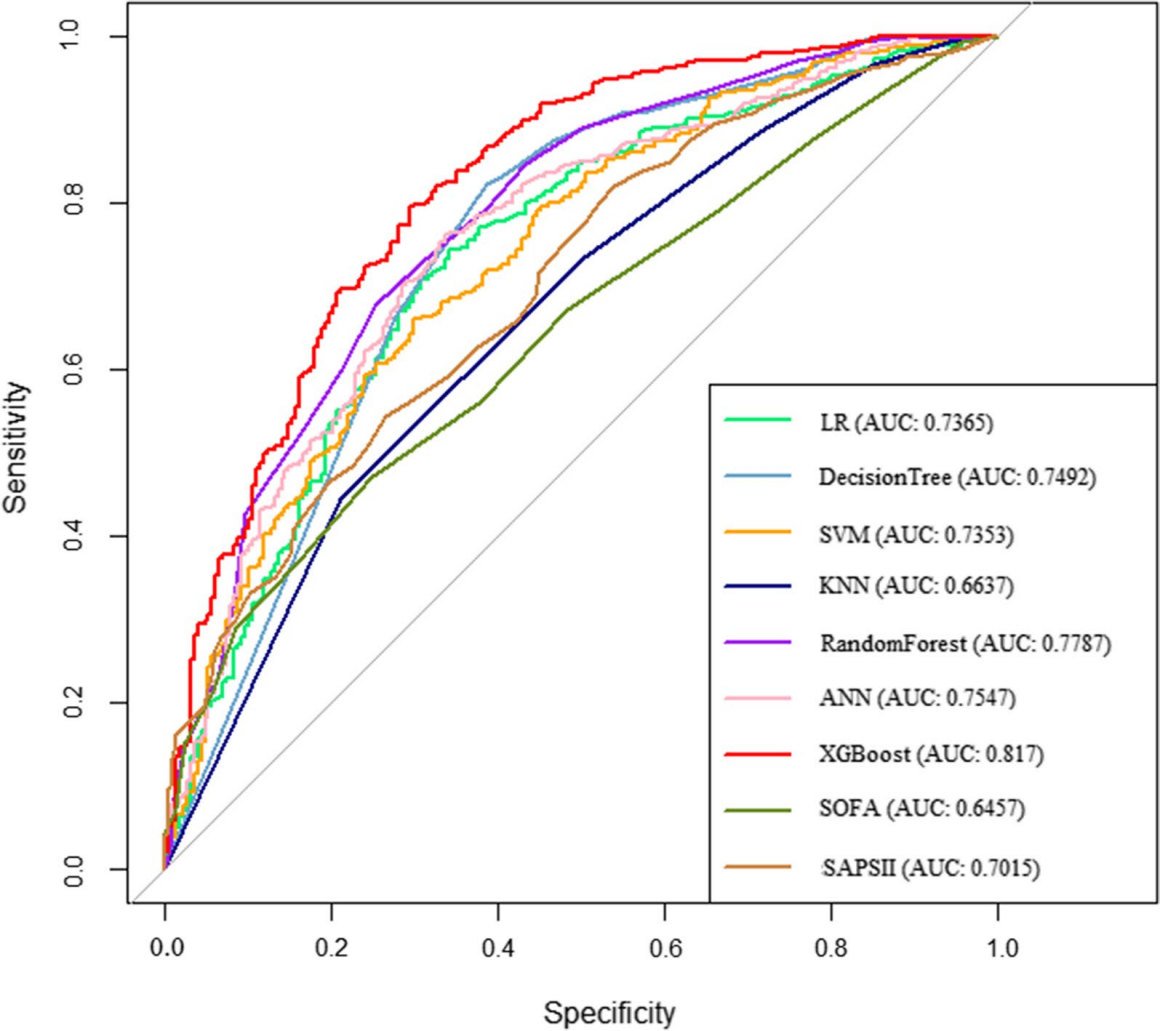
Receiver operating characteristic curve of the seven models. LR: logistic regression; KNN, k-nearest neighbors; SVM: support vector machine; XGBoost: Extreme Gradient Boosting; ANN: artificial neural network; SOFA: sequential organ failure assessment; SAPS II: the customized simplified acute physiology score; AUC: area under the curve

**Fig. 4:**
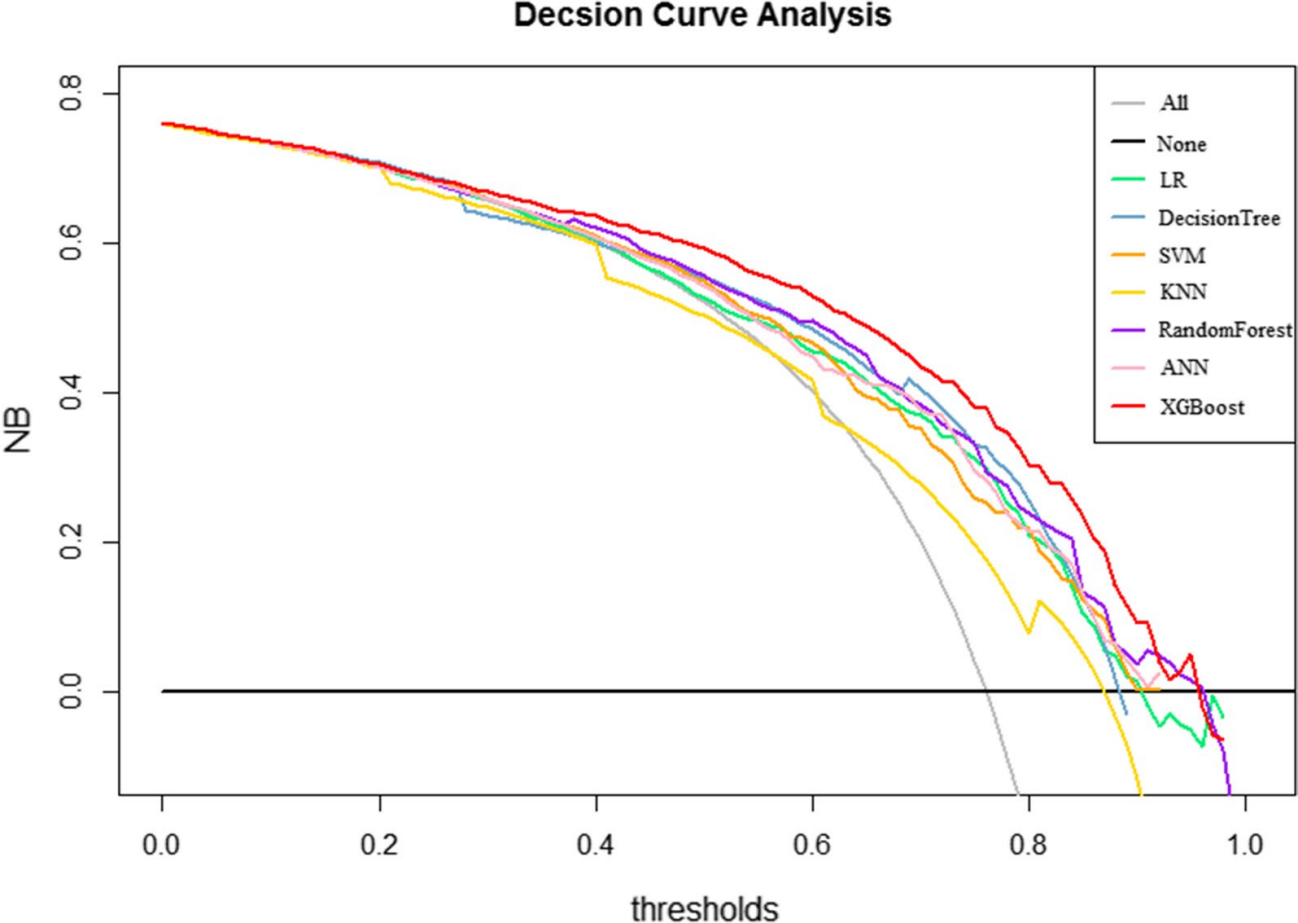
Decision curve analyses of the seven models. The horizontal line indicates no patients develop AKI, and the gray oblique line indicates patients develop AKI. *LR* logistic regression, *KNN* k-nearest neighbors, *SVM* support vector machine, *XGBoost* Extreme Gradient Boosting, *ANN* artificial neural network, *AKI* acute kidney injury

We generated seven ML models and two scoring systems to predict the development of AKI in patients with sepsis after ICU admission. Figure 3 shows the discriminative performance of nine models in terms of ROC curves. Among the nine models, XGBoost model (AUC = 0.817) had the best predictive effect for AKI in septic patients, followed by random forest (AUC = 0.779), ANN (AUC = 0.755), decision tree (AUC = 0.749), LR (AUC = 0.737), SVM (AUC = 0.735), SAPS II (AUC = 0.702), KNN (AUC = 0.664) and SOFA (AUC = 0.646) models. When using the LR model (AUC = 0.7265) as reference, the XGBoost model, random forest model, the ANN and decision tree outperformed it in the predictive ability of AKI in septic patients. However, the discrimination of SVM model (AUC = 0.735), KNN (AUC = 0.664), SOFA (AUC = 0.646), and SAPS II (AUC = 0.702) models were inferior to that of the LR model. Table 2 presents a set of detailed performance metrics for the nine models. The XGBoost model had the best discrimination with the highest sensitivity (0.945), accuracy (0.832), recall (0.852), F1 score (0.895), and the third highest specificity (0.913). In Additional file 1: Figure S1, the calibration curves showed that the XGBoost model performed best among the seven ML models. According to the DCA curves (Fig. 4), the XGBoost model exhibited greater net benefit along with the threshold probability compared with other models, indicating that the XGBoost model was the optimal model with favorable clinical utility.

**Table 2:** Model performance metrics

Models	AUC	Recall	Accuracy	F1 score	Sensitivity	Specificity
LR	0.737	0.796	0.765	0.858	0.834	0.878
KNN	0.664	0.798	0.742	0.840	0.886	0.857
SVM	0.735	0.797	0.788	0.874	0.833	0.926
Decision tree	0.749	0.834	0.793	0.870	0.910	0.882
Random forest	0.779	0.809	0.794	0.876	0.935	0.923
XGBoost	0.817	0.852	0.832	0.895	0.943	0.913
ANN	0.755	0.778	0.783	0.875	0.824	0.899
SOFA	0.646	0.755	0.723	0.781	0.633	0.712
SAPS II	0.702	0.774	0.762	0.814	0.811	0.845

*AUC* area under curve, *LR* logistic regression *KNN*: *k*-nearest neighbors, *SVM* support vector machine, *XGBoost* extreme gradient boosting, *ANN* artificial neural network, *SOFA* Sequential Organ Failure Assessment, *SAPS II* the Simplified Acute Physiology Score II

### Feature importance in XGBoost models

**Fig. 5:**
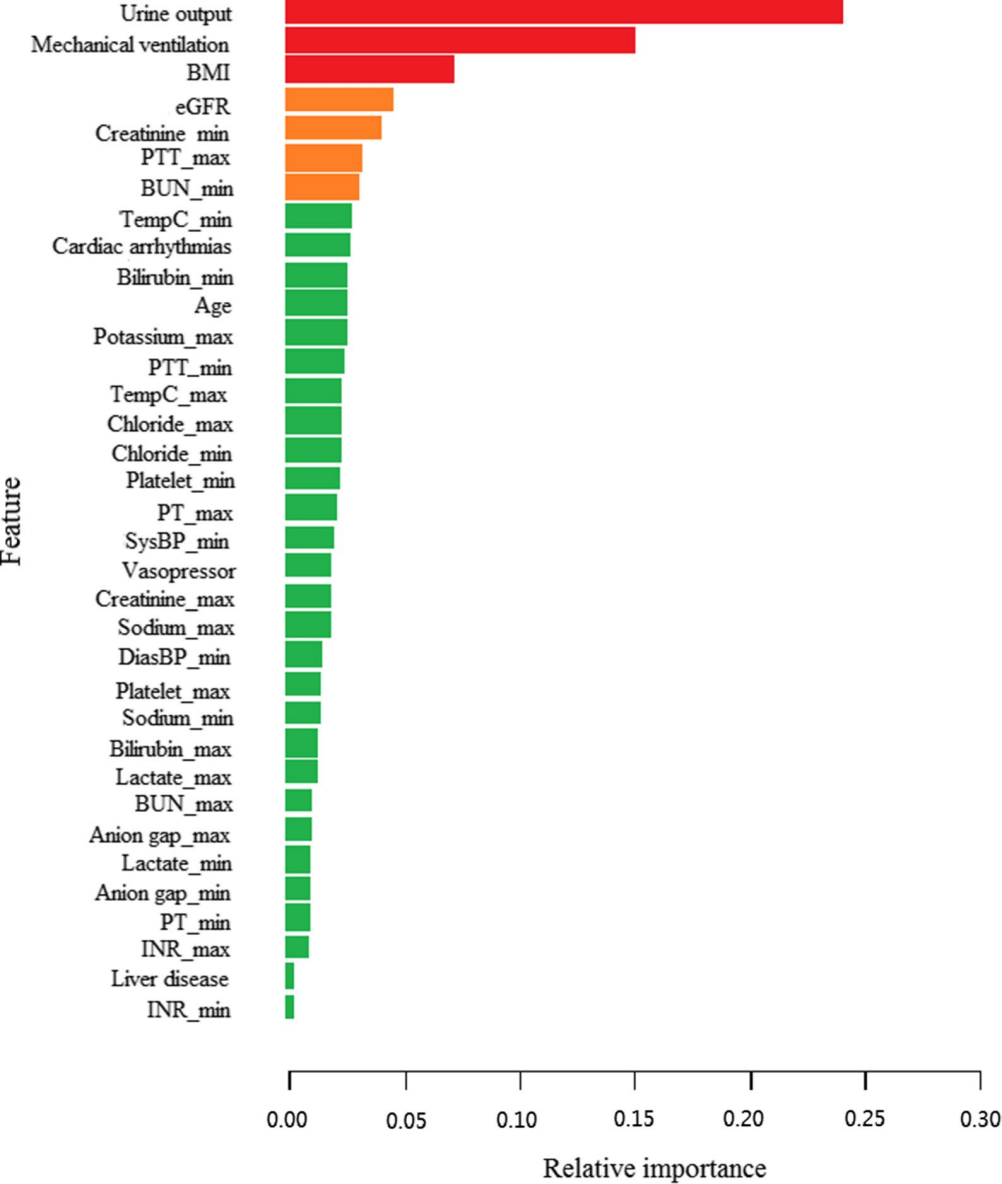
Feature importance derived from the XGBoost model. *BMI* body mass Index, *PTT* partial thromboplastin time, *BUN* blood urea nitrogen, *eGFR* estimated glomerular filtration rate, *Max* Maximum, *Min* Minimum, *AKI* acute kidney injury

The ranks of feature importance in the XGBoost model are shown in Fig. 5. Urine output, mechanical ventilation, BMI, eGFR, minimum creatinine, maximum PPT, and minimum BUN were the most important features that contributed to AKI in critically ill patients with sepsis.

## Discussion

Compared with some previous reports on AKI prediction in critically ill patients using the MIMIC- III dataset [18–20], our research has several novel contributions. For the first time, our study included seven commonly used ML algorithms for comprehensive analyses and compared their predictive performance with that of traditional scoring systems, including SOFA and SAPS II scoring systems. The ML models showed good predictive accuracy in term of discrimination and calibration, but it was not the same as usefulness in clinical practice. When the threshold probabilities of the net benefit are impractical, a model with good performance may also have limited applicability [27]. Therefore, we applied the DCA curves to validate the clinical applicability of the ML models. Finally, Boruta algorithm can help us fully understand the importance of independent variables, so as to carry out feature selection more effectively.

The incidence rate of sepsis is increasing in critically ill patients worldwide, which is associated with high mortality and economic burden [28]. Sepsis is a well-known risk factor of AKI, as the kidney is very sensitive to hypoperfusion and some interventions, such as mechanical ventilation and excessive fluid resuscitation. At present, the treatment of AKI in sepsis remains reactive and nonspecific, and no preventive treatment is available. The presence of AKI has a significant impact on increased mortality in septic patients, which range from 38.2 to 70.2% [29, 30]. Hernando et al. [31] found that AKI occurs in 40–50% of septic patients with a 6–eightfold increase in mortality. Furthermore, a prospective cohort study including 401 critically ill patients revealed that the incidence of AKI was 50.1% in patients with severe sepsis, which is 7.79 times higher than that in patients without sepsis [32]. However, active treatment at the early stage of AKI can improve the survival rate [33]. Some studies have found that early renal recovery in sepsis-related AKI can not only improve the survival rate, but also contribute to the later recovery of patients after discharge [34–36]. Unfortunately, it is difficult for clinicians to identify patients at high risk of AKI in the ICU. Therefore, developing and promoting reliable prediction models is particularly urgent for identifying these patients and providing them with timely and effective interventions to improve their prognosis.

In this study, the traditional severity scoring systems, such as SOFA and SAPS II scores, showed an unfavorable performance compared with the ML model, suggesting that they might not be effective tools for AKI prediction in critically ill patients with sepsis. Although SOFA and SAPS II scoring systems can be used to assess the risk of adverse outcomes in critically ill patients, these scores largely depended on the experience of the practitioners [37]. Moreover, these scoring systems preclude the analysis of a large number of valuable variables, resulting in a worse predictive performance than that of multivariate models [38]. Previous studies have revealed that SOFA and SAPS II scoring systems have some disadvantages, such as poor prediction performance, low sensitivity and specificity, wide fluctuation range, and cumbersome process, compared with ML models [16].

Our results showed that the XGBoost model had a better capability than the LR model for predicting AKI in septic patients. On the one hand, the LR algorithm requires researchers to manually select independent variables, cannot detect the complex nonlinear relationship and interaction between independent variable X and response value Y, and is sensitive to the multicollinearity of independent variables, which may result in an underfit and inaccurate model. On the other hand, the XGBoost model could efficiently and flexibly deal with missing data and combine weak prediction models to establish accurate prediction models. Due to its excellent precision and performance, the XGBoost algorithm is increasingly emphasized as a competitive alternative to LR analysis in predicting clinical adverse outcomes.

Among all ML models, the XGBoost model performs best in AKI prediction, which were consistent with some previous studies. Liu et al. [39] demonstrated that the predictive performance of the XGBoost model superior to three other ML models, including LR, SVM, and random forest, for predicting mortality in patients with AKI. Zhu et al. [40] found that the XGBoost model outperformed the KNN, LR, decision tree, random forest, and ANN models in prediction of hospital mortality for mechanically ventilated patients. Moreover, a meta-analysis revealed that XGBoost was more effective than LR and other ML algorisms, including ANN, SVM, and Bayesian network, in the prediction of AKI [41].

This study is the first to apply ML algorithms for predicting the development of AKI during hospitalization in patients with sepsis. Through the sophisticated XGBoost model, we identified that urine output, mechanical ventilation, BMI, eGFR, minimum creatinine, maximum PPT, and minimum BUN were mostly associated with the development of AKI in patients with sepsis. Among these features, urine output was considered to be the most important indicator of AKI, which is in accordance with the KDIGO recommendations. In addition to urine output, some measures of renal function, such as eGFR, BUN, and serum creatinine, also played an important role in the prediction of kidney disease. These results have been confirmed in many clinical studies. Mertoglu et al. [42] found that serum creatinine and BUN have greater diagnostic value compared with other novel markers including myo-inositol oxygenase and cystatin C. Laranja et al. [4] revealed that septic patients with AKI had lower urine output compared with patients with AKI from other cases or chronic kidney disease. Grams et al. [43] demonstrated that low eGFR was a reliable risk factors for AKI through a meta-analysis including more than 1 million participants from eight countries. Notably, mechanical ventilation was also significantly associated with AKI in septic patients. Positive-pressure mechanical ventilation (PPV) is commonly used in critically ill patients to provide oxygenation, ventilation, and airway protection support. However, PPV has long been considered to have potentially harmful effects on the kidney [30]. This may be due to the following three reasons. First, PPV may increase intrathoracic pressure and thus reduce venous reflux, cardiac output, and renal perfusion. Second, mechanical ventilation may induce the release of some neurohormones, affect the renin-angiotensin system, and decrease renal blood flow and eGFR. Third, mechanical ventilation at any volume or pressure might create a cascade of inflammation, including multiple interleukins, tumor necrosis factor-α, and Fas ligand, that may contribute to AKI. Moreover, PTT is common indicators to judge coagulation function. Recently, a retrospective study [44] showed that more than half of patients with septic AKI had at least one abnormal coagulation index, and coagulation dysfunction may predict poor outcome of patients. BMI is a simple and useful index for obesity according to the height and weight of patients. BMI has been widely studied in patients with sepsis and AKI. Our findings showed that the AKI group had higher BMI compared with the non-AKI group. Obesity can lead to glomerular hyperperfusion and hyperfiltration, increase the hemodynamic and metabolic burden of a single glomerulus, and activate inflammation of adipocytes and oxidative stress [45], increasing the risk and progression of AKI. As these indexes can be evaluated easily at hospital admission, they can be used as convenient predictors for the development of AKI in critically ill patients with sepsis.

In this study, the in-hospital incidence of AKI in septic patients was 75.5%, which was similar to some previous studies. According to the report by Fan et al. [15], the incidence rate of AKI was 61% in 15,508 patients with sepsis. Tejera et al. [32] conducted a retrospective study in 401 critically ill patients and found that the incidence of AKI was as high as 75.3%. The pathogenesis of AKI in sepsis is complex and has not been clarified yet. Hemodynamic instability, impaired endothelial function, infiltration of inflammatory cells in the renal parenchyma, renal thrombosis, and renal tubular necrosis have been hypothesized to contribute to the development of AKI in septic patients [46]. The hyperactivation of immune response caused by sepsis is particularly important in the pathogenesis process, including the proinflammatory and anti-inflammatory stages. In the proinflammatory stage, humoral and cellular immunity can cause a storm of inflammatory factors, leading to the excessive secretion of inflammatory factors (such as interleukin 1 and tumor necrosis factor-α), the activation of complement and coagulation system, the activation of hyaluronic acid and elastase, and eventually the reduction of renal blood flow and the occurrence of AKI and septic shock [6, 47]. Subsequently, patients will have a compensatory anti-inflammatory response, which is an immunosuppressive state, manifested by increased secretion of cytokines (such as interleukin 10), weakened endocytosis, reduced proliferation of lymphocytes, and increased apoptosis [29]. Thus, patients with sepsis are the high-risk group for AKI during hospitalization. Once AKI occurs, the prognosis is significantly worse, and even RRT cannot improve the prognosis.

We should acknowledge some limitations of this research. First, the retrospective and observational nature of our study may lead to inevitable selection bias. Second, the data of MIMIC- III came from a single center in the United States, which may affect the extension of the prediction model to other populations. Therefore, further research with large samples and multiple centers is necessary to externally verify the application of models. Third, we used the filling method to estimate some missing data, which may lead to deviation from the true value. However, we still believe that the constructed model is helpful for clinicians to timely treat ICU patients with sepsis at high risk for developing AKI.

## Conclusions

In conclusion, the ML models can be reliable tools for predicting AKI in septic patients. Among all of the predictive models, the XGBoost model is the most effective model, which may assist clinicians in tailoring precise management and implementing early interventions for septic patients at risk of AKI to reduce mortality.

## Supplementary Information

**Additional file 1: Figure S1**. Calibration curves of the seven models. The x-axis represents the predicted probability calculated by models, and the y-axis is the observed actual probability of AKI. *LR* logistic regression, *KNN* *k*-nearest neighbors, *SVM* support vector machine, *XGBoost* Extreme Gradient Boosting, *ANN* artificial neural network, *AKI* acute kidney injury.

## Data Availability

The datasets used and/or analyzed during the current study are available from the corresponding author on reasonable request.

## Abbreviations

AKI: Acute kidney injury
ICU: Intensive care unit
MIMIC-III: Medical Information Mart for Intensive Care III
XGBoost: EXtreme Gradient Boosting
LR: Logistic regression
KNN: *k*-Nearest neighbors
SVM: Support vector machine
ANN: Artificial neural network
SOFA: Sequential organ failure assessment
SAPS II: The customized simplified acute physiology score
ML: Machine leaning
CNNs: Convolutional neural networks
ICD-9: International Classification of Diseases and Ninth Revision
KDIGO: The Kidney Disease: Improving Global Outcomes
RRT: Renal replacement therapy
SpO_2_: Oxygen saturation
BMI: Body mass index
SysBP: Systolic blood pressure
DiasBP: Diastolic blood pressure
PTT: Partial thromboplastin time
PT: Prothrombin time
INR: International normalized ratio
BUN: Blood urea nitrogen
TempC: Temperature
WBC: White blood cell
Vent: Ventilation
Max: Maximum
Min: Minimum
eGRF: Estimated glomerular filtration rate
AUC: Area under curve
ROC: The receiver operating characteristic curves
OR: Odds ratio
CI: Confidence interval
DCA: Decision curve analysis
PPV: Positive-pressure mechanical ventilation
