# Supplementary figures and images for: Machine learning for the prediction of acute kidney injury in patients with sepsis
Source: J Transl Med. 2022 May 13;20:215. doi: 10.1186/s12967-022-03364-0 (PMC9101823; doi:10.1186/s12967-022-03364-0)

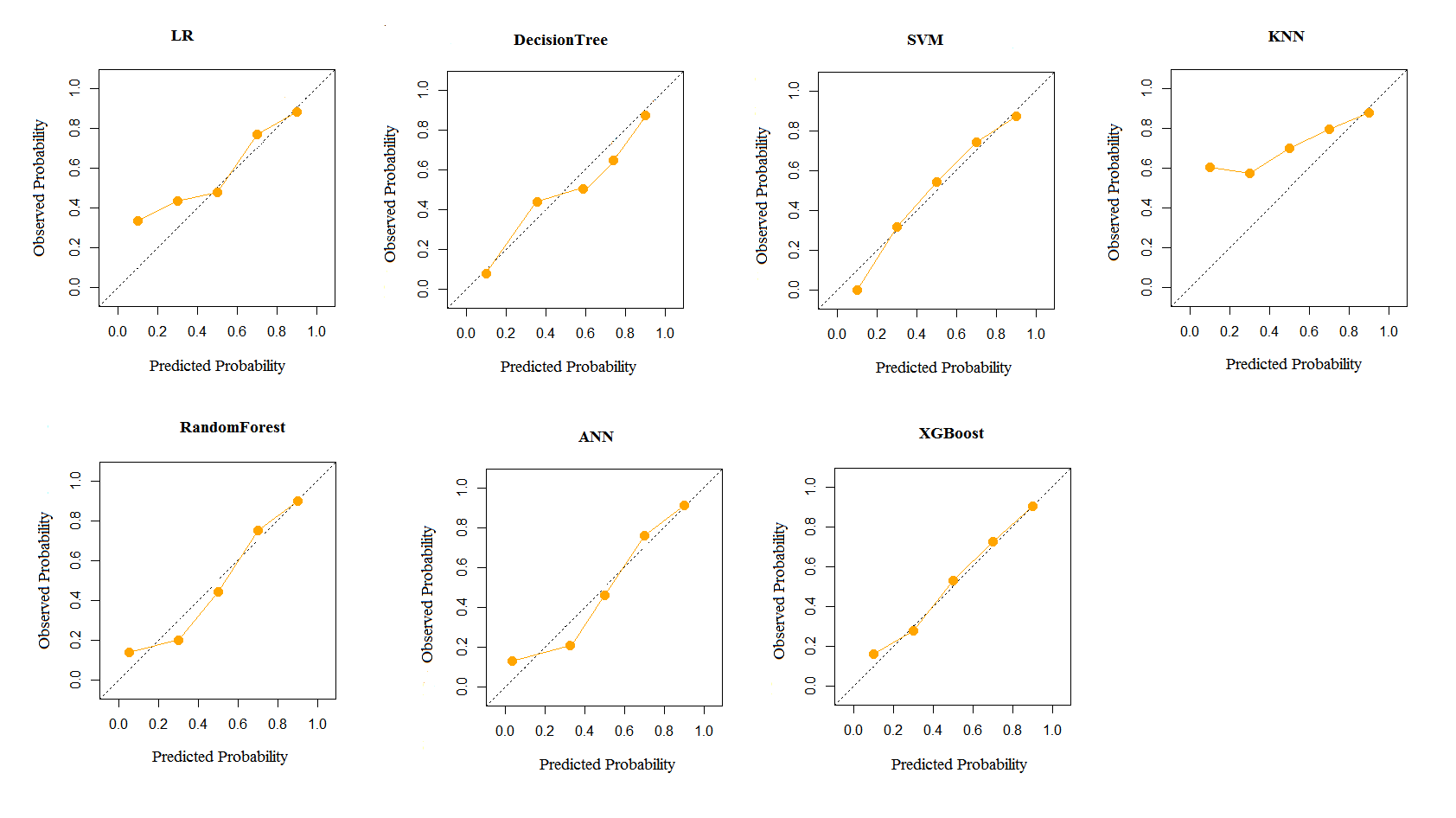

Supplement: Supplementary file 1 — Additional file 1: Figure S1. Calibration curves of the seven models. The x-axis represents the predicted probability calculated by models, and the y-axis is the observed actual probability of AKI. LR logistic regression, KNN k-nearest neighbors, SVM support vector machine, XGBoost Extreme Gradient Boosting, ANN artificial neural network, AKI acute kidney injury. [file 12967_2022_3364_MOESM1_ESM.tif]
